# Outpatient education, a momentous in clinical education: a qualitative study of medical students’, faculty members’, and residents’ perspectives

**DOI:** 10.1186/s12909-023-04694-3

**Published:** 2023-10-03

**Authors:** Marziyeh Barzegar, Seyed Aliakbar Faghihi, Mitra Amini, Nahid Zarifsanaiey, Elham Boushehri

**Affiliations:** 1 Clinical Education Research Center, Department of Medical Education, School of Medicine, Shiraz University of Medical Sciences, Shiraz, Iran; 2 E-Learning Department, Virtual School, Shiraz University of Medical Sciences, Shiraz, Iran; 3 Department of Medical Education, School of Medicine, Hormozgan University of Medical Sciences, Bandar Abbas, Iran

**Keywords:** Medical education, Ambulatory education, Outpatient, Medical students

## Abstract

**Background:**

Even though a lot of scholars have been looking at outpatient education lately because it has become more popular and they want to know about its successes, failures, and problems, we have not been able to find a complete study. Therefore, our study aims to gain a deeper understanding of the various aspects of outpatient education based on the actual experiences of medical students, faculty, and residents.

**Methods:**

Face-to-face and semi-structured interviews were used to collect data for this qualitative study. Until data saturation was reached, the interviews continued. A total of 21 participants from Shiraz University of Medical Sciences, including medical students, residents, and teachers, were enrolled. The Guba and Lincoln-first written standards for scientific accuracy in qualitative research were used to figure out how reliable the data were.

**Results:**

Fourteen categories were extracted from four main themes. The results show that four categories: “physical space and equipment,” “prerequisites related to the curriculum,” “teaching skills development,” and “near-peer teachers” should be considered for outpatient education preparation. Theme 2, “implementation requirements,” included “student dimension,” “faculty’s commitment to planning,” and “program supervision.” Theme 3, “challenges of outpatient education,” was described by five related categories, including “curriculum implementation challenges,” “student challenges,” “faculty challenges,” “system-related challenges,” and “patient-related challenges.” Finally, two categories emerged about facilitators of outpatient education: internal and external facilitators.

**Conclusion:**

Outpatient clinics represent a crucial aspect of medical practice. To effectively leverage this resource, preliminary planning, considering all the prerequisites, paying attention to the implementation requirements, getting to know the challenges, and trying to solve them, especially with incentives, are essential.

## Background

Academic lectures, ward rotations, and outpatient education comprise the majority of clinical medical education. Outpatient education has become increasingly popular in recent decades for two reasons. Firstly, they are more effective in improving medical students’ learning; secondly, teaching rounds of inpatients are no longer as effective as they used to be [1, 2].

In the past decade, numerous adjustments have been made to the healthcare system. One of the greatest changes has been moving away from traditional inpatient care and toward ambulatory care [1, 3–5]. Inpatient cases are less reflective of the average clinical case presentation because they more frequently involve critically ill patients or patients with specialized illnesses [1, 3, 4, 6, 7]. In most wards, medical students are usually assigned to examine previously diagnosed patients with a set treatment plan [1, 7]. This highlights the significance of clinical education in ambulatory care settings. What occurs in a clinic is comparable to what a physician would encounter in his or her office [6, 8]. In ambulatory clinics, students can experience various disorders under common conditions [1, 8, 9]. Furthermore, ambulatory care clinics are crucial for acquiring and developing students’ clinical skills, such as history-taking, clinical reasoning, physical examination, chronic disease management, and interprofessional collaboration [1, 2, 6, 8, 10, 11].

This has prompted numerous professional societies, such as the “American College of Physicians (ACP),” the “Society of General Internal Medicine (SGIM),” and the “American Association of Medical Colleges,” as well as accrediting bodies, such as the “Liaison Committee on Medical Education and the Higher Learning Commission,” to advocate for the expansion and further development of ambulatory education [1, 12, 13].

Outpatient education, despite its importance and benefits, faces many challenges and is sometimes criticized as a dying art. Recent research has highlighted many difficulties in ambulatory care clinics that can significantly impair the educational experiences of both medical students and faculty members [2].

Many scholars have recently focused on outpatient education due to its increased prominence and subsequent curiosity about its achievements, failures, and obstacles. These studies have primarily been carried out in the context of program evaluation via assessing learning [9] or from the viewpoint of one of the stakeholders, students [6, 8, 14], or directors [12]. Although some researchers have studied the challenges confronting this education [1, 2], and others have tried to develop models for enhancing the quality of education [5, 15], a comprehensive study does not appear to exist based on our search. Consequently, our study aims to develop a deeper understanding of the various aspects of outpatient education based on the actual experiences of medical students, faculty, and residents. By involving all three groups in the research, we attempted to identify every aspect of ambulatory education in Iranian universities of medical sciences to provide educators and curriculum developers with a means to make the necessary adjustments and enhance the quality of education in this setting. Specifically, the following research questions were sought to be answered in this study:

- Question 1: What are the prior conditions for establishing and implementing outpatient education based on participants’ viewpoints?
- Question 2: What are the implementation aspects of outpatient education based on stakeholders’ viewpoints?

## Methods

### Study design

This study used a qualitative approach and conventional content analysis to describe a phenomenon. By resolving “how” and “why” research questions, this type of study can yield rich and varied insights into individual experiences [16]. The qualitative content analysis aims to comprehend the phenomenon under investigation better [17]. We utilized qualitative research methods to further understand how participants’ experiences shaped their meaning and how that changed over time. Content analysis can provide reliable outcomes, generate insights, and set a path for organizational activities from textual data [18].

### Participants

Participants were medical school faculty members with teaching responsibilities in the university-affiliated clinics. In addition, students and residents with experience in outpatient education were chosen from a range of clinical levels. As clinical education for medical students takes place in the final three years and at different levels, we aimed to maximize variation in our sample by including clinical semester students from different admission years. Exclusion criteria consisted of participants’ refusal to continue cooperating.

Purposeful sampling was employed, and sampling continued until data saturation, at which point no additional information was gathered regarding the concept.

A total of 21 medical students, residents, and faculty members of Shiraz University of Medical Sciences were enrolled and then interviewed (Table 1).

**Table 1:** Demographic characteristics of the interviewees

P	Academic ranking	Gender
S1	Medical student, semester 11	Male
S2	Medical student, semester 14	Male
S3	Medical student, semester 12	Female
S4	Medical student, semester 13	Male
S5	Medical student, semester 14	Female
S6	Medical student, semester 11	Male
S7	Medical student, semester 11	Male
S8	Medical student, semester 10	Male
S9	Medical student, semester 12	Female
F1	Professor of Interventional Cardiology	Male
F2	Associate Professor of Nephrology	Male
F3	Professor of Surgery	Male
F4	Assistant Professor of Gastroenterology and Hepatology	Male
F5	Assistant Professor of Dermatology	Male
F6	Assistant Professor of Gastroenterology and Hepatology	Female
F7	Associate Professor of Neurology	Female
R1	Medical Resident of Dermatology	Female
R2	Medical Resident of Cardiology	Female
R3	Medical Resident of Internal Medicine	Male
R4	Medical Resident of Obstetrics and Gynecology	Female
R5	Medical Resident of Surgery	Male

### Data collection

In-depth, semi-structured interviews in Persian were conducted over eight months, from January 2022 to August 2022, with the permission of the administration of the medical school. Individual interviews were conducted for each participant. The experiences of the participants were elicited following the study’s objectives. From the beginning of their clinical rotations up to the time of the interview, all participants were asked to recall their experiences. For instance, if an interview took place with a student at the end of their twelfth semester, questions would cover their whole experience from the tenth to the twelfth semester.

The time and location of the interviews (in a private room in a hospital, clinic, or school where the study was conducted) were arranged with participants who agreed to participate.

A general question was initially posed during the interview, and as the interview progressed, the questions were steered toward the research objective. The interview started with a general, open-ended question: “Please describe a day from your most recent outpatient education experience.” Based on the responses, we asked participants the following in-depth questions to gain a deeper understanding of the concept under investigation: “What are a faculty member’s duties and responsibilities in a clinic with students’ attendance?” “How is learning assessed in this setting?” and “What are the most challenging issues?” In-depth questions were also posed, such as “Can you clarify further?” “Describe more,” and “You said that … please explain?” At the end of every interview, I asked questions such as, “Do you have any ideas or comments?” “Do you think there is a question that has not been addressed?” and “Would you like to question me?”.

All interviews were recorded with the participant’s permission. The first researcher with the requisite training for conducting qualitative studies, conducted and transcribed interviews. Only the first researcher knew the participants’ identities, while other researchers received interview transcripts that had been anonymized. The mean duration of the interviews was 42.24 min, ranging between 25 and 80 min, based on the circumstances of the interviewees and their assent.

### Data analysis

Graneheim and Lundman’s qualitative content analysis method was used to analyze the data by identifying meaning units, codes, subcategories, categories, and themes [19]. During the research period, data collection and analysis were performed simultaneously. The interviews were meticulously transcribed immediately following each interview by the first author, and each transcription was read multiple times to achieve data immersion and gain a general comprehension of the interview. After the interview, the interviewee was contacted to clarify any uncertainties.

Two researchers (MB and EB) then read each text line by line, identified meaning units, and assigned codes to each meaning unit. MB and EB read the preliminary codes multiple times and then contrasted them to identify similarities and distinctions. In the event of disagreement, a third researcher (AF) was involved. Next, codes were assigned to subcategories based on their similarities and differences. Similar subcategories formed categories, and then themes emerged (representing the text’s hidden meaning).

### Trustworthiness

To assess the trustworthiness of the collected data, the Guba and Lincoln-first published standards for scientific accuracy in qualitative research were applied: credibility, confirmability, dependability, and transferability [20]. For credibility, the researcher spent a significant amount of time immersed in the data and transcripts, and the allotted time for accumulating and analyzing the data helped the authors obtain a deep insight into the data. Different stakeholders (medical students, residents, and faculty members) with varying levels of expertise in each group were chosen as part of a triangulation technique for confirmability. In addition, a subset of participants was shown the preliminary data codes, and categories to acquire their views and feedback (member checking). To obtain a consensus, we discussed any contentious points to reach a final agreement.

An independent observer’s opinion was sought to assess the dependability of the findings. The external observer was a researcher with experience in both qualitative research techniques and outpatient education who was not a part of the research team. The consistency of the results was validated by this external review.

To ascertain the dependability, an external observer’s opinion was solicited. A researcher with experience in outpatient education and qualitative research techniques served as an external observer who was not a part of the research team. The consistency of the results was validated by this outside review.

A thorough explanation of the study’s concepts, participants, content, data collection, analysis methods, and limitations confirmed the data’s transferability. This will allow other researchers to replicate the study’s procedures.

### Translation of qualitative data

Qualitative research relies on translation to accurately convey participants’ meanings across languages, maintaining its trustworthiness. The literature suggests back-translation, consultation, collaboration, and piloting to solve translation problems. Each method has pros, cons, and costs.

Consultation is a practical approach to addressing the risk of losing meaning.

In the ideal translation process, it is essential to recognize that translation should not be viewed as a single, completed task upon receiving the translated materials from the translator. Consultation entails discussions between a researcher proficient in research methods and a translator knowledgeable in language and culture [21]. In this study, the researcher (EB) provided a bilingual translator with an overview of the research context and language to create consensus on the translation approach. Translation dilemmas were resolved through a three-way interaction involving the interviewers (MB and AF), the researcher (EB), and the translator, enhancing translation accuracy.

### Ethical considerations

The ethical approval for the study was acquired from the Research Ethics Committee of Shiraz University of Medical Sciences (IR.SUMS.REC.1400.456). All methods were carried out in accordance with relevant guidelines and regulations or the Declaration of Helsinki. Participants were given detailed information about the study’s goals before interviews were conducted, and their signed informed consent was obtained before any interviews were recorded. The anonymity of the participants, their responses, and the use of aliases or codes in quotations were guaranteed. Participation in the research was entirely voluntary and would not interfere with their academic progress, and they were given the option to drop out at any time. The interview and encoding data were encrypted before being saved to a personal hard drive for long-term storage. We promised to do so if a participant wanted to see the findings as a group.

## Findings

Out of 21 participants, seven were faculty members, nine were medical students, and the remaining were residents. The experiences and perceptions of participants were extracted and analyzed. Based on the results of the analysis, 14 categories were extracted within four main themes after coding and comparing the codes based on similarities and differences: “preparation for outpatient education” (Table 2), “outpatient education implementation requirements” (Table 3), “challenges of outpatient education,” and “facilitators of outpatient education.”

**Table 2:** Categories and subcategories of Theme 1

Theme	Category	Subcategory
**Preparation for outpatient education**	Physical space and equipment	
	Prerequisites related to the curriculum	elective programs
		learning objectives, and educational content
		instructional strategies
		supplementary resources
		patient as an educational case
		organizing student/ patient/ faculty contact models
	Teaching skills development	from experiences
		from teacher training programs
	near-peer clinical teachers

**Table 3:** Categories and subcategories of Theme 2

Theme	Category	Subcategory
**Outpatient education implementation requirements**	student dimension	students’ familiarization
		students’ assessment
	faculty’s commitment to planning	
	program supervision	

### Theme 1. preparation for outpatient education

The theme “preparation for outpatient education” refers to what should be considered and what preliminary planning should be done before implementing an outpatient education program. Based on the participants’ experiences, this concept pertains to the four basic components: physical space and equipment, prerequisites related to the curriculum, teaching skills, and near-peer clinical teachers (Table 2).

#### Physical space and equipment

This indicates that in designing a successful educational clinic, we should pay attention to the number of rooms, the connection of rooms, the quantity and quality of examination equipment, the number of examination beds and chairs.

*“It seems that three rooms are necessary for each clinic; a clinic that is large or includes several departments has enough space for students to move between the rooms, see examinations, and even rest.”* [S7].

#### Prerequisites related to the curriculum

Before implementing a curriculum, it is important to consider several factors based on the participant’s experiences to organize the program efficiently. These factors, as subcategories, are listed below.

##### Elective programs

The clinical elective course can be a valuable, highly regarded experience with benefits in providing better learning and academic success and gaining clear insight into their future postgraduate specialty. According to interviewee perceptions, some specialized and sub-specialized clinics can be considered locations for elective programs.

“*It happened to me that I went to the ophthalmology clinic and saw the patients next to the teacher, and I thought this field was very interesting, but because my attendance was optional, not mandatory.*” [S1].

##### Learning objectives and educational content

Learning outcomes and expected skills must be defined to devise a program that makes the most of the learning opportunities for the students. According to the participants’ perceptions, student learning opportunities in outpatient clinics usually focus on history-taking, physical examination, pharmacology, drug prescription, clinical approach, interpretation of paraclinical data, counseling the patient’s family, patient education, and professionalism.

“*The faculties insist, and it is true in my opinion, that the student takes a history first; usually, the students do a clinical examination after the history taking.”* [R2].

*“In my opinion, drug prescription is very important. Because, when we graduate, we must prescribe these drugs ourselves. In the clinic, the teacher’s approach to the patient is very important. That is, how does he diagnose, how does he manage, and how does he follow up?”* [S3].

Besides, learning objectives should genuinely prepare students for the demands of professional practice.

*“In fact, I do a needs assessment based on what skills my students will need when they become a doctor.”* [F3].

##### Instructional strategies

A program needs to have an efficient and dynamic set of teaching tools to be effective. According to the experiences of participants, a variety of strategies can be applied: a brief overview of the disease shortly before the patient enters, structured observation, active and gradual student participation, content delivery from simple to complex, consideration of differences in the knowledge level of learners, supervision and feedback, practical teaching, lecturing, conferences, and debriefing.

“*Always, in the clinic, before a patient comes in, I talk to the students about that patient for 5–10 min and overview the disease.”* [F4].

*“In my teaching sessions, I first determine my audience. If they are students, interns, residents, or fellows, I tailor my goals, vision, and educational content accordingly.”* [F5].

*“Sometimes, teachers provide us with conferences that feature common clinical cases.”* [S2].

*“We review the patient’s history with the teacher, who provides additional explanations as needed. I keep track of feedback to address any issues.”* [S3].

##### Supplementary resources

Experience has shown that readily available additional resources or instructional aids can be used to enhance learning. These resources, based on interviewees' experiences, include a summary of the cases of that day, laboratory reports and radiographs of patients, and internet-based resources.

*“As part of our course, I instruct students to access dermatology atlases online.”* [F2].

##### Patient as an educational case

Learning in clinical settings heavily relies on patients as a valuable resource. Participants believed that an ideal volume of patient encounters, pertinent to learning objectives and expected qualifications of a doctor, is important.

*“During the week, if I have patients suitable for learning, I postpone their appointments to a day when my clinic is educational.”* [F1].

*“In the tumor clinic, only tumor cases are visited, and if I, as a GP, am going to see a few tumor patients, this does not truly help me.”* [S9].

*“In clinic A, the number of admitted patients was low, and we could easily communicate with the patient and receive training.”* [S2].

The appropriateness of the number of patients compared to the number of students in a clinic was also highlighted.

*“In a certain clinic, the number of interns and students was small, allowing us to examine each patient for at least 20 min.”* [S4].

##### Organizing student, patient, and faculty communication models

A variety of different models for organizing student, patient, and teacher interactions in outpatient clinics have been described, according to the participants.

*“I use different models. Sometimes, we visit the patient together, and the student observes. Sometimes, the students visit the patient alone, and then they present the important points of the patient to me.”* [F1].

#### Teaching skills development

To achieve competence in teaching, faculty members must acquire knowledge and skills in educational principles. Participants’ experiences reflected the view that the teaching competencies of faculty members can be achieved through their experience over time or through formal teacher training programs.

*“A teacher gains appropriate experiences over time, which improve his/her teaching skills.”* [F7].

*“Training can help faculty members be better teachers.”* [F4].

#### Near-peer clinical teachers

It is known that residents play an important role in teaching undergraduate medical students, and based on the experiences of most participants, the resident performs his/her teaching role better if this role is defined as a duty and commitment in the educational system.

*“If residents understand their teaching roles from the beginning and take responsibility for educating, it will improve our education.”* [S3].

### Theme 2. outpatient education implementation requirements

Based on the participants’ experiences, the concepts of implementation requirements refer to the three basic components. The first two relate to the students’ and faculty’s commitment to planning, and the last is program supervision (Table 3).

#### Student dimension

Actions related to students must be taken while present in the clinic for effective learning. This includes familiarization and assessment.

##### Students’ familiarization

According to the participants’ statements, a briefing session on the initial day of each clinic is indispensable.

*“From the very first day of the course, our teachers informed us about the topics that would be taught, the conferences that would be held, and the presenters who would be delivering the lectures. This helped us immensely.”* [S5].

Students’ readiness for apprenticeships or internships was affected by how clearly their duties and expectations were outlined.

“*What I wanted to say is that it should be clear what they want from me, that is, when I come in the morning, I should know what I should do to be in the norm and what I should not do. Well, this increases productivity, in my opinion.”* [S1].

##### Students’ assessment

According to participants’ experiences, a continuous assessment of students’ clinical skills is important for switching from a static to a dynamic education.

*“On-the-spot assessment can be more effective than evaluation at the end of a three-month internship. The teacher may not even remember who I was or what I did. In any case, I went to the clinic today and spent 2 or 3 h there, so how I performed should be considered.”* [S6].

#### Faculty’s commitment to planning

Based on participants’ statements, it refers to allocating enough time to teaching and the teacher’s management skills.

*“Some teachers extended the training period to work with fewer patients, leading to more detailed examinations and benefiting both the patient and our learning.”* [S7].

*“A faculty member in a clinic is not just a teacher but must manage her environment; for example, he or she should make sure that the clinic is not crowded, patients are visited on time, students are satisfied, have a scheduling secretary, and no one is late.”* [F2].

#### Program supervision

A list of supervisory considerations must be considered to minimize errors and resolve problems. These include teacher performance, student attendance, and patient quantity and quality.

*“In terms of the educational system, there should be a difference between a teacher who spends time teaching students and a teacher who does not.”* [S4].

*“Sometimes, the student would leave the clinic or not participate without anyone noticing. This is not acceptable.”* [S8].

*“Of course, it is necessary to give feedback to the teacher about his performance…”* [F4].

*“Someone, somewhere, must specify that a clinic supposed to be educational only accepts up to 10 patients*[S3].

### Theme 3. challenges of outpatient education

It refers to barriers that interfere with valuable and effective education in clinics. This theme can be described in five related categories, including curriculum implementation challenges, student challenges, faculty challenges, system-related challenges, and patient-related challenges.

#### Curriculum implementation challenges

It refers to factors affecting curriculum delivery. Inappropriate implementation of near-peer clinical teaching was identified as one of the program's challenges. Participants defined turbulence as incompatible integration of trainees, lack of resident commitment to teaching due to lack of sleep and long shifts, and lack of knowledge and self-confidence to teach students.

*“When residents and fellows are present in the clinic simultaneously as students, we do not receive any teaching. The cases suitable for residents or fellows are boring for students.”* [S8].

*“A resident is a person who hasn’t slept for 48 h, hasn’t drunk a drop of water, hasn’t eaten, and has been struggling for the past 12 h. How can such a person be expected to teach.”* [S2].

*“My experience is that I should not trust what the resident says. I don’t know how much they read from reference books and how much is based on experience; their working time is long and, they don’t have time to update their knowledge.”* [S5].

One of the problems with designing and implementing the educational tool was the lack of involvement from all stakeholders, as well as the logbook being reduced to just a tool for roll calls.

*“We did not use a log book. On the last day of the rotation, we would go to one of the residents, get his stamp, and stamp all the activities, of course, if there was a log book.”* [S9].

*“In most departments, only one person creates the logbooks by copying and pasting from other sources.”* [F3].

Reducing students’ educational duties to the level of secretarial work, prioritizing the health system over education, and the small share of outpatient education in total clinical education were other stated challenges emphasized by most participants.

*“We and sometimes residents are responsible for filling out forms and recording documentation without prior guidance.”* [S7].

*“There were many times when I felt like the teacher was assigning us menial tasks instead of teaching.”* [R2].

*“According to one of our clinical teachers, our top priority should be the health system and patient care. If time allows, we can also focus on medical education, which is secondary to our primary goal.”* [S4].

*“Our clinical education mostly takes place in the hospital, with very limited outpatient education opportunities.”* [S1].

Several students also mentioned patients with specialized conditions as a noteworthy challenge.

#### Student challenges

It refers to problems that students struggle with. One of the most critical obstacles raised by the participants in this category was related to unsystematic and irregular student assessment. These unsatisfactory experiences include lack of a plan or objective criteria for student assessment.

*“Outpatient education lacks a specific assessment program. Students are typically evaluated at the end of each department rotation.”* [F3].

*“They only tell us that we will give you a score out of 20. It is not clear on what basis they give this score.”* [S6].

In turn, student assessment has been limited to students’ attendance.

*“During an evaluation, the clinic secretary asks attendees to raise their hands. This becomes their score at the end of the course.”* [S9].

The incongruity of the student assessment method with the educational program or content was another challenge stated by participants.

*“Most of our assessments are only theoretical, even though the internship training is practical, but they take a multiple-choice exam, with questions more related to teaching in the inpatient ward than in the clinic.”* [S5].

The students were unhappy that the resident assessed them without being involved in their teaching.

*“The score of the students’ exams is often in the hands of the residents, while the residents do not teach. Some students try to maintain their relationship with the resident to get a better score.”* [S2].

Other issues, such as lack of awareness of learning objectives, lengthy clinic duration, student fatigue, and neglect of student welfare, were declared along with the challenges of student assessment.

*“During the teaching session, we must be on our feet; we don’t have time to drink water or go to the bathroom. In terms of comfort, I would like to point out one simple thing: the presence of a chair. We must stand for at least three hours. In such cases, we do not pay attention to the learning. In the last clinic I went to, we were on our feet for four and a half hours; I could not feel my legs, and it did not have a learning aspect.”* [S6].

Eventually, the students complained about feeling invisible in clinics and uncertain about their future job prospects.

#### Faculty challenges

The presence of supportive teachers can significantly improve trainees’ experiences in the clinic. However, outpatient clinician-educators face several challenges.

The diversity and multiplicity of the roles of academic staff, a lack of motivation (inadequate financial and spiritual incentives), and a lack of commitment to teaching were issues stated by all three participant groups.

*“A faculty member is expected to be a therapist, teacher, publisher, administrator, and make economic demands. This workload can reduce their motivation and make them feel like robots."* [S4].

Scant knowledge and skills and inadequate or low-quality teacher training programs in outpatient teaching were other challenges.

*“Many teachers taught us based on their experiences as students, and they did not know how to teach in a clinic.”* [S7].

*“Faculty development programs are often in the form of lectures and are not taught very practically.”* [F7].

Based on some students’ views, little knowledge or not up-to-date or evidence-based knowledge of faculties in the specialized and professional field of medicine was a serious issue.

*“We felt that the teacher was not scientifically up-to-date, which was why he did not answer our question.”* [S9].

A lack of feedback and supervision on student performance was also highlighted.

*“We were taking the patient’s medical history, but the teacher was not paying attention. I may have made some errors in my history taking, and there was no one to provide feedback or guidance on improving.”* [S1].

Another challenge was the lack of skills to communicate safely and without anxiety with students and residents.

*“In certain fields, teachers humiliate residents and interns to the point where they cannot sleep due to fear.”* [S6].

#### System-related challenges

Institutional policies fail to support outpatient education due to a lack of resources, incentives, and evaluation criteria for clinician educators.

*“The university (system) looks at students and us as cheap labor; practically, the burden of the system (hospital) is on our shoulders. If we want to go to the clinic to be taught, then who will do the work of the hospital?”* [R5].

*“When the research activities of a faculty member are evaluated, an h-index is used. His/her therapeutic activities are based on the number of patients visited, operations performed, *etc*., so how are his teaching activities measured? or where are they included at all?”* [S3].

#### Patient-related challenges

In this category, we describe two main issues related to patients as one of the main components of clinical education that can pose challenges. One of them is the difficulty of respecting the patient’s privacy.

*“In the clinic, there are typically ten students for every patient. It can be difficult for a patient to discuss personal problems, such as a perirectal abscess.”* [S1].

The other challenges are the excessive number of patients and their specialized conditions in most clinics.

*“One day, 80 patients were visited in a clinic within 5 h; we could not see or hear anything anymore.”* [S8].

*“In the tumor clinic, only tumor cases are visited, and if I, as a GP, am going to see a few tumor patients, this does not really help me.”* [S9].

### Theme 4. facilitators of outpatient education

Two categories emerged regarding facilitators of outpatient education: internal and external facilitators. It refers to factors that can facilitate learning and teaching in outpatient education, improve effectiveness, and be associated with greater student and faculty satisfaction.

#### Internal facilitators

The internal factors affecting outpatient education refer to the subjective preferences, contentment, and pleasure experienced by an individual. Individuals acquire or impart knowledge without expecting rewards such as grades or commendations. In expressing the participants’ experience, students’ personal interest in learning was one of the issues that emerged*.*

*“My motivation to learn, even if the teacher isn’t willing to teach, drives me towards becoming a good doctor.”* [S4].

*“In my opinion, a significant aspect of teaching is personal; a teacher must possess an intrinsic concern for education. Teachers devote enough time based on their conscience and personality.”* [S3].

Faculty members’ positive personality traits, such as compassion, friendliness, and humor, in their relationships with students and patients are among three factors that are related.

*“The teacher had a very intimate and warm relationship with the patient and us; he joked, which made us more interested and involved in the issues.”* [S1].

*“She taught and listened not only to student criticisms and suggestions, but also to their stories, which greatly aided learning.”* [R3].

The faculty’s personal interest in teaching and desire for self-improvement were other components.

*“I believe the teaching profession should only be pursued by those with a positive attitude towards education. Otherwise, the prevailing mindset is to prioritize healthcare and treatment.”* [F6].

#### External facilitators

Participants in the current study identified external factors affecting outpatient education, including faculty salary support, clinician educator job promotion, bonuses, teacher training programs, and accommodations for patient volume.

*“Part of the difference in the quality of teachers’ teaching is related to the system. I mean, the amount of support the system gives to the faculty influences his or her quality of performance.”* [R5].

*“Schools must manage the teacher’s teaching time in such a way that the financial loss of the teacher is compensated, for example, to give a special privilege to teaching, either financially or professionally.”* [F2].

## Discussion

This research aimed to identify all the facets of outpatient education. In addition, including three sets of stakeholders was a crucial part of the study’s methodology. The present research results revealed the difficulties and factors that might assist outpatient education, in addition to the concerns before and during its implementation.

One of the prerequisites for the successful implementation of outpatient education is a clinic with adequate space and minimal necessary equipment [22]. Despite this, there is no established standard for the equipment and accommodations an academic outpatient clinic should have [2, 23], and a lack of suitable sites has been cited as a source of dissatisfaction in numerous studies [12, 24–29].

Electives, which can be taken in addition to the core curriculum, allow students to tailor their education to “their specific requirements and/or interests” and develop a comprehensive insight into their intended postgraduate field. Therefore, this issue should be given some consideration in the development of the curriculum.

This study identified two fundamental steps in curriculum development approaches: clarifying objectives and related content and identifying educational strategies.

Like some other studies, this study indicates that various patient management skills can be improved within the outpatient setting [10, 23, 26, 30–33].

According to the findings of this study, various teaching techniques can be employed to enhance learning in outpatient settings. The decision about which methods to use is extremely complex and influenced by factors such as student knowledge level, learning objectives, as mentioned by our participants, teacher experiences, educational funding, facilities, and equipment [34, 35]. Using textbooks and researching educational materials before patient visits are also valued by trainees [10].

Although no conclusion about the optimal case mix for learning can be drawn from studies to maximize educational efficiency [23], appropriate patient selection is essential [36, 37]. The number of patients seen per session will affect patient education and care [23, 38].

The choice of a contact organization model for students/patients depends on the number of faculties, available rooms, and present students. However, instructors should always emphasize the importance of students’ engagement in their learning [30].

Clinical faculty members are expected to teach medical students without adequate training. Faculty members often improve their teaching abilities through personal experiences and observing skilled teachers [36, 39–41]. According to some participants’ perspectives, it is a trial-and-error process that, for novice teachers, usually leads to errors.

Within the teaching interaction, it is essential to contemplate the role of the near-peer teacher. In several countries, including the United States, the United Kingdom, and Canada, all residents are expected to undergo training to teach. The potential benefits of training all residents at an institution are significant and emphasize the importance of a teaching position there [42].

In this study, the outpatient education implementation requirements included three categories: student dimension, faculty commitment to the planning, and program supervision.

As a “golden rule,” orientations were held at the onset of each clerkship rotation to review clerkship objectives, schedule activities, and establish performance expectations [27, 43, 44].

This study found that formative assessment is as crucial as summative assessment in evaluating and improving student learning. During practical instruction, formative assessment can be conducted using a predefined evaluation grid [9], a log book [5, 45], and self-assessment progress [46, 47].

Although the program’s effectiveness depends on the time commitment of faculty, clinic, and patients, time constraints are always a challenge [2, 11, 12, 15]. As highlighted in this study, a faculty member must possess educational management and leadership competencies to fulfill the diverse stakeholders’ expectations effectively [42].

In the “program supervision” category, participants revealed that conducting a systematic investigation of various facets of the program is necessary. The Ambulatory Care Learning Education Environment Measure (ACLEEM) questionnaire has been suggested as a valid and reliable instrument to determine the efficacy of an ambulatory education program and identify areas for improvement [4, 6, 43, 48, 49]. This study identifies outpatient education challenges, including curriculum implementation, student, faculty, system, and patient-related challenges. Understanding these challenges can help improve future programs.

In curriculum delivery, problems such as inappropriate incorporation of various levels of trainees and difficulties with developing and implementing the logbook were revealed. Although logbook implementation in outpatient education has been mentioned in some studies, its use and efficacy have not been evaluated [5, 38, 50–52]. According to this study, the main obstacles to near-peer clinical teaching are residents’ lack of interest or aptitude for teaching, clinical knowledge deficiencies, and their demanding workload. This is supported by research on "resident as teacher" studies [42, 50, 51]. The prioritization of health systems over medical student education is also emphasized [10, 52].

The assessment methodology employed in the clinic posed the most significant challenge for the students. Limited student engagement in the learning process and their passive (shadowing) role, resulting in fewer chances for independent learning, are additional hindrances confirmed by other studies [1, 4, 15, 28].

This study, like others, has identified various obstacles that outpatient clinician-educators encounter. Balancing their teaching responsibilities with other roles [11, 15], the use of ineffective teaching methods, insufficient ambulatory teaching skills, a lack of interest or commitment to teaching [1, 2, 12], as well as inadequate teacher feedback and supervision on student performance [1, 2, 9, 11, 12, 15, 28], were among these challenges. Lack of skills to communicate with students and residents and not having evidence-based knowledge in the professional field of medicine in some cases were identified as additional concerns.

The challenges related to the system include a need for more support for clinician educators [11, 15], a shortage of healthcare human resources addressed by involving students and residents, and an absence of quantifiable criteria to assess the educational role of educators.

Managing patient volumes in clinics is never easy [1, 28, 53]. It seems that patient selection for educational reasons may address both this problem and the specialization of patients [9]. Although important, safeguarding patient privacy can be a complex matter that is not always easily resolved.

Based on this study, faculty’s and student’s self-interest facilitated teaching and learning. In this study, internal and external motivational factors were positive personality traits of teachers, self-improvement or development through a formal faculty training program, and supporting clinician educators via salary and job promotion. Some of these factors have been considered influential in other studies [2, 12, 28, 41].

Although not directly mentioned in our study as a necessity for setting goals, curriculum planning, and curriculum delivery, social accountability must be considered in reviewing of the outpatient curriculum for relevance to cultural context as well as community, national, and regional priority health needs [54].

This study has limitations, such as time-consuming research and difficulty scheduling interviews with busy faculty members. Because the study used purposive sampling, the research findings cannot be generalized to a larger population.

## Conclusion

Outpatient clinics represent a crucial aspect of contemporary medical practice. Given the growing prevalence of patients in this setting, medical education endeavors must cultivate educational prospects that effectively leverage this resource. Merely arranging for students to participate in this setting does not provide a comprehensive solution. Effective outpatient education begins with proper preparation and consideration of prerequisites before the students enter the field. During the implementation, supervision of various program aspects will help to improve it. At the same time, being aware of the potential challenges of the program and paying attention to external and internal motivators will increase the chances of its success and efficiency.

## Data Availability

The datasets used and/or analyzed during the current study are available from the corresponding author on reasonable request.
